# Extreme behavioural and psychological symptoms of dementia: a case study

**DOI:** 10.1186/s12888-024-05785-1

**Published:** 2024-05-10

**Authors:** Megha Mulchandani, Agatha Conrad

**Affiliations:** 1 Hunter New England Mental Health Service, PO Box 833, Newcastle, NSW, 2300 Australia; 2 Older People’s Mental Health Service, Hunter New England Mental Health Service, Waratah, NSW, Australia; 3 Healthy Minds Program, The University of Newcastle, Callaghan, NSW, 2308 Australia

**Keywords:** Older people, Treatment, Dementia

## Abstract

**Background:**

The seven tiered behavioural and psychological symptoms of dementia (BPSD) model of service delivery has been used by inpatient units. The classification of each tier is broadly defined and not always agreed upon by clinicians. The case study uses novel approach by combining the BPSD classification criteria with clinical presentation to identify the clinical characteristics of the case and match these characteristics against the BPSD classification. This process was enhanced by using case specific measures such as the Neuropsychiatric Inventory (NPI) and Cohen Mansfield Agitation Inventory (CMAI) scales and key clinical data.

**Case Presentation:**

A case study of 76 year old male diagnosed with mixed Alzheimer’s and Vascular dementia. The clinical presentation of the symptomatology was deemed to be extreme, thus fitting into the seventh tier (Extreme) of the BPSD model of service delivery. The case is considered to fit into the Extreme BPSD category given the high levels of aggression, which were consistently reflected in high scores on NPI and CMAI, as well as long length of inpatient stay (over 3 years). The average number of Pro re nata (PRN) psychotropics medications per month was 56 and seclusion episodes of 6 times per month, with each episode lasting on average 132 min shows severity of behaviours. His level of aggression had resulted in environmental damage and staff injuries.

**Conclusion:**

We recommend patient clinical characteristics, relevant hospital data and specific measures should be used to develop consensus around defining and classifying cases into Extreme BPSD.

## Background

Dementia, is also known as a Major Neurocognitive disorder, and is defined by a significant decline in cognitive function which impairs capacity to perform everyday activities independently [1]. Dementia is typically diagnosed based on a combination of clinical history, brain imaging, screening blood tests and cognitive screening tests or more detailed neurocognitive testing.

In addition to the diagnostic symptomology, dementia is often accompanied by BPSD. This is a broad umbrella term that encompasses various neuropsychiatric symptoms which include agitation, aberrant motor behaviour, anxiety, elation, irritability, depression, apathy, disinhibition, delusions, hallucination, sleep or appetite changes [2]. The estimated rates of BPSD are variable but have found to be to up to 99% of the population with dementia [3, 4].

**Fig. 1:**
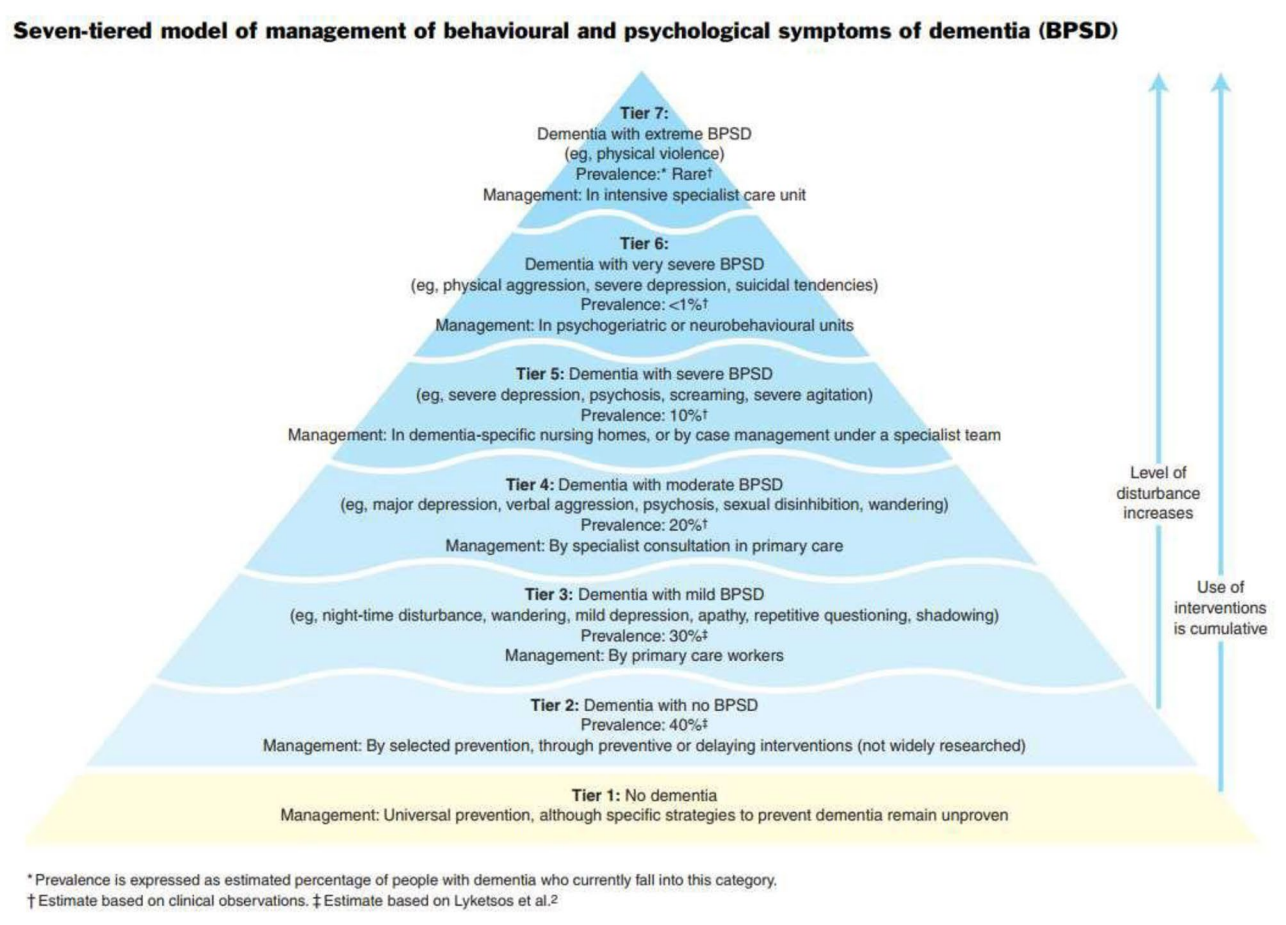
Seven tier BPSD model for service delivery

As shown in Fig. 1 below, Brodaty, Draper & Low (2003) proposed a seven tiered BPSD model for service delivery based on severity of symptoms and prevalence of BPSD, with tier 1 defined as those with no dementia and those is tier 7 are defined as having Extreme BPSD from high levels of unprovoked violent behaviour towards other residents and staff [5].

Individuals within Tier 7 are usually of younger age (under 70), male with a robust physique. The type of dementia is often non-Alzheimer’s (e.g.: vascular dementia, alcohol related brain damage, frontotemporal dementia). Due to the significant and specialised care needed in these circumstances, such people require high security specialist care unit [5].

Since the original concept of seven tiered BPSD model for service delivery was introduced, little work has been done to define each of the tiers, including ‘Extreme BPSD’. Not surprisingly, the interpretation of ‘Extreme BPSD’ symptoms are subjective and based on clinical experience and exposure. There is a possibility that clinicians may misclassify people in lower tiers into Extreme BPSD based on clinical presentation [6].

Anecdotally, within our unit, staff found it difficult to classify patients into the correct tier based on clinical presentation alone. In addition to patient clinical characteristics on presentation, we decided to include specific measures to quantify the behaviour as well as hospital data to allow for more accurate definition and classification of extreme BPSD. The current case study describes a patient with diagnosed dementia who has been classified as Extreme BPSD based a combination of patient clinical characteristics, hospital data and specific measures.

## Case presentation

Mr X is a 76-year-old Caucasian male, with a diagnosis of mixed Alzheimer’s and Vascular dementia, who was a patient of our acute mental health inpatient service for 3 years, with ongoing significant levels of both verbal and physical aggression.

His background was defined by a diagnosis of Post-Traumatic Stress Disorder (PTSD) (combat related and complex childhood trauma) and major depression. He was also a domestic violence perpetrator. There was developmental delay as a child and his family have described him premorbidly as someone who was quick to temper.

Mr X’s medical history included Meniere’s disease with cochlear implant, benign prostate hypertrophy, asbestosis, Type II Diabetes Mellitus, Hypertension. There was history of syphilis exposure with patient self-report of treatment; further test results were not consistent with latent or neurosyphilis. He had a history of multiple head injuries, with loss of consciousness but whether this resulted in any cognitive deficits could not be ascertained through history. There was no history of epileptic seizures.

He was an ex-smoker and had a history of alcohol use which was reported to be heavy for approximately 4 years, many decades ago. There was no past history of illicit substance use. There was no family history of dementia, and no personal forensic history. Mr X was of average intellectual functioning who completed tertiary education and worked in the armed forces but retired medically due to PTSD.

Concerns around Mr Xs’ cognition, short- and long-term memory impairment, began 6 years ago when he was still living with his wife. The first assessment was conducted about 5 years ago where he scored 23/30 on Roland Universal Dementia Assessment Scale cognitive screening test. Approximately a year later he scored 21/30 on Mini Mental State Examination and 67/100 on Modified Mini-Mental State. A plain CT brain showed mild prominence of ventricles and sulci and mild chronic small vessel ischaemia within the periventricular, deep and peripheral white matter. CT SPECT revealed mild to moderately reduced perfusion in the occipital, temporal, parietal and frontal cortices. All screening blood test results were essentially normal apart from mild anaemia and low Vitamin D (33 nmol/L, range 50–140). It was around this time, about 5 years ago that he was diagnosed with mild to moderate Alzheimer’s dementia.

During this period and before coming into our service for the current admission, Mr X was admitted twice to acute older people’s mental health unit, first time when he was first diagnosed with dementia, and subsequently expressing suicidal and homicidal ideation, complicated by cognitive decline with persecutory and grandiose delusions, He was treated with Haloperidol 5 mg daily, Memantine 15 mg, Quetiapine 400 mg total daily dose, his symptoms stabilised and then was discharged home. However, his mental health continued to deteriorate, and he was re-admitted to the acute older people’s mental health unit, and then discharged to an aged care facility.

During the first five months in the aged care facility, he continued to be verbally and physically aggressive towards staff, resulting in an involuntary admission to our acute older people’s mental health unit. On assessment, he was observed to be experiencing psychosis with delusional content relating to other ethnic community and alien invasion from which he needed to be cleansed. On mental state examination, he was described as a tall well-groomed and dressed gentleman who was calm and seen to be smiling and happily greeting people in the department. His speech was of normal tone, and volume but noted to have some word finding difficulty. His mood was “pretty good” and his affect was reactive. His thought form was described to be disorganised with limited coherent responses to questions. No perceptual disturbance was noted but he admitted to experiencing nightmares relating to his time in the armed forces. He was noted to be disorientated to time and place, but not to person, and he scored 1 out of 4 on clockface drawing. His insight was partial, and he had some recollection of the aggression at the aged care facility but was unable to comprehend the ramifications of his actions. As part of the assessment, blood tests and CT scan was done, which were normal and in keeping with previous results.

During the entirety of his admission Mr X continued to display aggression, complicated by PTSD. His care required a high nursing ratio and low stimulus environment. This environment is staffed by experienced nursing staff, under the care of the Psychogeriatrics team and supported by specialised allied health team. As part of the treatment, multiple medication changes were made including change from Memantine to Rivastigmine 9.5 mg/24 hour. Other medications such as Sodium Valproate and Carbamazepine trials to therapeutic range did little to change behaviour. Prazosin was cross titrated with reducing doses of Quetiapine, again with little change in presentation. Fluoxetine to 20 mg showed some reduction in dissociative episodes.

Pain, constipation, sleep and infections were managed with a combination of pharmacological and non-pharmacological interventions. Intense work was done to develop a behaviour support plan which detailed his behaviour at every stage of the behavioural agitation scale from calm to extreme aggression.

Despite the intense care environment, with specialised multidisciplinary care, Mr X continued to display significant levels aggression, both verbal and physical. Whilst some were due to medical reasons (delirium), most were due to either psychological or environmental factors. Even minor environmental alterations would lead to agitation or aggression, such a change in his nursing special (from morning to afternoon shift) or socially unacceptable behaviour by other patients sharing his space, and noise or malfunctioning cochlear equipment.

His presentation was further complicated by use of rapid tranquillisers which were used to contain physical aggression, but instead resulted in increased confusion, exacerbating agitation and aggression. Seclusion was used a means of de-escalation and avoidance of parental rapid tranquillisers, and ultimately Mr X care was transferred to Mental Health Intensive Care Unit (MHICU). Mr X has remained in MHICU for the over 2 years and during the entirety of this period Mr X’s care involved 1:1 nursing special during his waking hours.

As an expected part of his diagnosis, Mr X showed both cognitive and functional decline over the period of his three-year admission.

There were two project specific measures used to monitor impact of intervention on Mr X behaviour (1) Neuropsychiatric inventory assess to assess the frequency and severity of symptoms and level of disruptiveness [7]; (2) Cohen-Mansfield Agitation Inventory which measures the level of aggression/agitation [8].

Neurodegenerative disorders were assessed by nursing staff using the Neuropsychiatric inventory (Nursing home version), consisting of 10 neuropsychiatric symptoms/domains rated on a frequency and severity scales. The frequency of symptoms is rated on a Likert scale from 1 to 4, with 1 indicating “rarely – less than once a week” and 4 being “ very often – essentially continuously present”. The severity of symptoms is rated from 1 to 3, with 1 being” mild – changes in appetite or eating are present but do not lead to changes in weight loss and are not disturbing” 3 – being “severe - obvious changes in appetite or eating are present and cause changes in weight, leading to weight loss which may upset the resident” [7]. The total score is calculated by multiplying frequency by severity for each domain, then adding the 10 domains together to get a total score. The total score can range from 10 to 120. In addition, each symptom/domain is rated in terms of occupational disruptiveness which measures how much does this behaviour negatively impact the staff or carer or creates extra work for staff or carer, The symptoms/domains are rated on 0 to 5 Likert scale with 0 being “not at all” and 5 being “very severely or extremely disruptive" (very disruptive major source of distress for staff and other residents, requires time usually devoted to other residents). The scores for occupational disruptiveness range from 0 to 50 [7].

The Neuropsychiatric inventory was administered on two separate occasions to Mr X by nursing staff on the unit. On the first occasion Mr X scored 67 (44 on frequency and severity and 23 points for occupational disruptiveness) and two months later, Mr X score increased to 98 (62 for frequency and severity, with 36 points for occupational disruptiveness).

The measured Cohen-Mansfield Agitation Inventory (CMAI) is a carer (staff) questionnaire. The 29 behaviours seen in dementia are rated for frequency – the lack of focus on severity is corrected by the breadth of behaviours covered. The behaviours covered include verbal aggression, repetitiveness, screaming, hitting, grabbing and sexual advances. The Cohen Mansfield Agitation Inventory (CMAI) was administered on two separate occasions two months apart, Mr X scored 90 on the first occasion and 106 on second occasion. Mr X tended to display aggressive behaviour towards staff most of the time in a day and even several times in an hour.

Over the 3-year period Mr X received numerous pharmacological interventions to manage his behaviour with an average of 56 PRN psychotropic medications per month to manage agitation and aggression. Seclusion was used as the main non-pharmacological intervention to manage risk to others and minimise the use of psychotropic medications. Over the 3-year period, Mr X was secluded on average 6 times per month, with each episode lasting on average 132 min. Furthermore, there have been over 400 incidents logged for physical and/or verbal aggression.

Attempts were made to gain data on staff injury (including time lost to work) and environmental damage, however, records were not kept on a central database. Anecdotally, Mr X’s level of aggression had led to significant damage to his environment, such as breaking doors off hinges. He had also assaulted staff resulting in shoulder and wrist injuries leading to time off work.

## Discussion and conclusions

There is no widely agreed objective measures to quantify the behaviour and accurately classify the severity of BPSD. The current classification of extreme BPSD is broad in describing the behaviours in terms of high levels of unprovoked violent behaviour towards other residents and staff [8]. It was often difficult for staff to precisely define what the high level of unprovoked violent behaviour is and therefore staff didn’t feel as confident in using this classification model of care. The two measures chosen for the project, helped to quantify the violent behaviour in terms the level of disruption (NPI) and aggression (CMAI) towards staff and other patients. The scores from NPI and CMAI measures together with a patient clinical characteristics, and hospital data allowed staff to more confidently and accurately classify the severity of BPSD. The crucial clinical characteristics that were considered as part of the classification of BPSD, included a history of PTSD, alcohol abuse and domestic violence, as well as complex medical history.

He was robust male of a younger age group with difficulties in verbal communication due to his dementia. The level of severity, frequency and intractability of BPSD was evidenced by the length of admission in an acute inpatient environment with a 1:1 nursing special. Attempts to manage BPSD through various combination of high doses of psychotropics led to limited improvement in agitation and aggression. This approach was combined with a detailed tailored behavioural support plan and was regularly updated to meet his changing clinical needs.

The clinical history and observational data together with project specific measures were supplemented by more routinely collected hospital data. The high levels of disruptive behaviour and aggression when measures were collected was mirrored by the hospital data particularly the lengthy admission (over 3 years), number of PRN medications (average 56 medications per month) administered,  and number of seclusion episodes (6 times per month). The combination of data allowed staff to confidently and accurately classify this case into the extreme BPSD.

However, we have only anecdotal evidence on injury to staff and damage to property which is a limitation in this case study. We were unable to quantify psychological impact of aggression on other patients and staff. We would recommend that incident data pertaining to injuries to staff, patients and environment is collected routinely to provide a more complete picture and a stronger definition and classification of extreme BPSD.

## Conclusion

We described in detail clinically, further supported by clinical data of a gentleman whose behaviours qualify for Tier 7 of the Brodaty et al., (2003) triangle. We found that it was the combination of patient characteristic, hospital data and specific measures that help staff to more accurately classify the case. We recommend patient clinical characteristics, relevant hospital data and specific measures should be used to develop consensus around defining and classifying cases into Extreme BPSD.

## Data Availability

The data that support the findings of this study are available from Hunter New England Local Health District but restrictions apply to the availability of these data, which were used under license for the current study, and also are not publicly available. Data are however available from authors upon reasonable request and permission from Hunter New England Local Health District. For any enquiries regarding data availability please contact Dr Megha Mulchandami, Megha.Mulchandani@health.nsw.gov.au.

## Abbreviations

BPSD: Behavioural and Psychological Symptoms of Dementia
NPI: Neuropsychiatric Inventory
CMAI: Cohen Mansfield Agitation Inventory scale
PRN: Pro re nata
